# Environmental Heavy Metal Contamination from Electronic Waste (E-Waste) Recycling Activities Worldwide: A Systematic Review from 2005 to 2017

**DOI:** 10.3390/ijerph18073517

**Published:** 2021-03-29

**Authors:** M. G. Karel Houessionon, Edgard-Marius D. Ouendo, Catherine Bouland, Sylvia A. Takyi, Nonvignon Marius Kedote, Benjamin Fayomi, Julius N. Fobil, Niladri Basu

**Affiliations:** 1 Regional Institute of Public Health Comlan Alfred Quenum, University of Abomey-Calavi, Ouidah 384, Benin; eouendo@yahoo.fr (E.-M.D.O.); kedmar@yahoo.fr (N.M.K.); 2 School of Public Health, Université Libre de Bruxelles, 1070 Brussels, Belgium; catherine.bouland@ulb.ac.be; 3 School of Public Health, University of Ghana, Accra LG 13, Ghana; phykles@gmail.com (S.A.T.); jfobil@gmail.com (J.N.F.); 4 Occupational Health and Environment Research Unit, University of Abomey-Calavi, Cotonou 06 555, Benin; bfayomi2@yahoo.fr; 5 Department of Natural Resource Sciences, McGill University, Montreal, QC H9X 3V9, Canada; niladri.basu@mcgill.ca

**Keywords:** e-waste, heavy metals, soil, water, sediment, environment

## Abstract

The recycling of electronic waste (e-waste) contaminates ecosystems with metals, though a compilation of data from across sites worldwide is lacking, without which evidence-based comparisons and conclusions cannot be realized. As such, here, a systematic review of the literature was conducted to identify peer-reviewed studies concerning e-waste sites (published between 2005 and 2017) that reported on the concentration of heavy metals (Cd, Hg, As, Pb and Cr) in soil, water and sediment. From 3063 papers identified, 59 studies from 11 countries meeting predefined criteria were included. Reported metal concentrations were summarized, and a narrative synthesis was performed. This review summarized 8286 measurements of the aforementioned metals in soils (5836), water (1347) and sediment (1103). More than 70% of the studies were conducted in Asia. In nearly all cases, the average metal concentrations in a particular medium from a given site were above guideline values; suggesting soils, water and sediment at, or near, e-waste recycling sites are contaminated. Across all media, concentrations of Pb were generally highest, followed by Cr, As, Cd and Hg. The synthesized information demonstrates that e-waste sites worldwide are contaminated with metals, that geographic data gaps exist, that the quality of most studies can be improved and that action is needed to help reduce such levels to protect human health and the environment.

## 1. Introduction

The generation of electronic waste (e-waste) has rapidly emerged to become of growing concern worldwide [1,2]. For example, in 2019, the global community generated 53.6 million metric tons of e-waste, equivalent to 7.3 kg per person of e-waste per year, compared to 5.8 kg/person in 2014. By 2030, the amount of e-waste is expected to reach 74.7 million metric tons [3]. According to 2014 estimates, the largest e-waste producer was the United States (which produced 7.1 million tons), followed by China (which generated nearly 6.0 million tons). Regarding per capita generation, countries in Europe generated the most e-waste, averaging 15.6 kg of e-waste per person [4]. However, disparities have been observed between countries that produce e-waste and those that receive it. For example, there are estimates that China, India and some African countries receive up to 80% of global e-waste [5]. This exemplifies the disproportionate flow of e-waste from high-income countries to developing ones [6,7] even though we note that, in 2018, China introduced legislation to stop the import of e-waste into its territory [8].

Activities within the entire e-waste sector are notorious for contaminating ecosystems with a range of potentially toxic elements [9], as documented in several case studies from around the world [10,11,12,13,14,15]. This contamination of ecosystems is linked, among other things (such as processing and recycling), to the fact that e-waste continues to be dumped near rivers and deposited in illegal landfills [6,16]. Contaminants in e-waste include heavy metals known to be hazardous to environmental and human health [12,17,18,19,20,21,22,23,24,25,26,27]. It is important to note that the risk of contamination is more concerning in developing countries given that much of the work is practiced within the informal sector, unlike in, for example, Europe and North America where modern approaches and safety rules are instituted [3,28].

Despite the growing literature on ecosystem contamination at e-waste sites, there is a lack of a worldwide review on the subject matter from which we can synthesize global understanding, perform comparative studies (e.g., across sites or against guidelines), identify data gaps and ultimately draw evidence-based conclusions and make recommendations. Notably, a recent review by the WHO and the United States National Institute of Environmental Health Sciences—U.S. NIEHS [29]—called out a need for better exposure measurements at e-waste sites, and particularly a synthesis of evidence on a worldwide basis given the tremendous variation that exists across locations. While some reviews on the subject matter have been conducted, they have targeted specific regions or countries [12,17,30,31,32], instead of trying to understand the worldwide situation. Thus, the objective of the current study was to perform a systematic review of the literature to identify peer-reviewed studies that reported on the concentration of heavy metals (Lead—Pb, Mercury—Hg, Cadmium—Cd, Arsenic—As and Chromium—Cr being amongst the most common and concerning in such sites [3]) in environmental media (soil, water and sediment) from e-waste sites worldwide. In doing so, this systematic review is intended to increase overall understanding of ecosystem contamination at e-waste recycling sites.

## 2. Methods

### 2.1. Search Strategy

A systematic review method was developed based on guidance from the United States Office of Health Assessment and Translation [33]. A literature search using MEDLINE and Web of Science (on 7 February 2018) was conducted using the following parameters: ((metals OR “heavy metal” OR mercury OR Hg OR Lead OR Pb OR chromium OR Cr OR cadmium OR Cd OR Arsenic OR As)) AND (((Sediment OR soil OR water)) AND ((((E-waste) OR “electronic waste”) OR WEEE) OR (“waste electric and electronic equipment”))).

### 2.2. Study Selection Criteria

**Figure 1:**
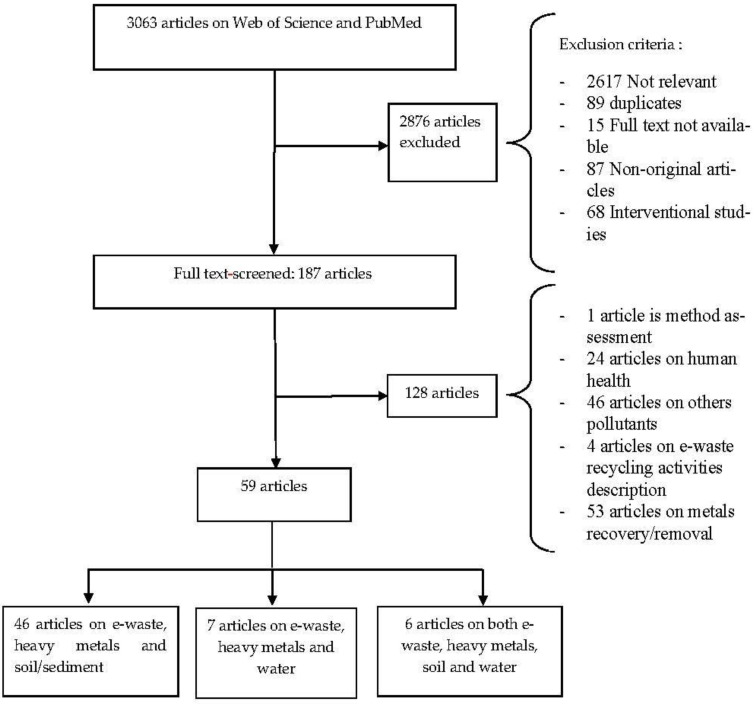
PRISMA flow diagram indicating the number of articles that were identified, screened and included in the current review. PRISMA = Preferred Reporting Items for Systematic Reviews and Meta-Analysis.

The scientific papers were reviewed in a two-step process (Figure 1): first, the title and summary fields were queried for relevance, and second, the full texts were examined for articles that were considered potentially relevant. We focused our search to include articles that were original primary scientific studies (as opposed to reviews) that had abstracts available in English or French (which reflected the authors’ backgrounds). We also focused on exposure assessment studies and did not include works that were principally focused on health outcomes or methods development. For studies to be included, we needed to access the full paper, and the work had to provide an estimate of the central tendency value and a measure of the variation from which an upper limit could be estimated. When a study was the subject of several articles, we chose the article containing the most complete dataset to serve as a representative piece. The scientific research studies included were carried out over the period of 2005–2017.

### 2.3. Data Extraction and Analysis

From each included paper, data were collected on the study design (sampling location, time, type of sample and sample size), measurement (units of measurement, technology for metal detection, detection limit, accuracy and precision), the type of heavy metal (As, Hg, Cr, Cd or Pb) and the environmental media (soil, water and sediment), and the results were measured as both a central tendency value (geometric mean or median or mean) and a high-end value (95th or 90th or 75th percentile or maximum value).

The different units of measurement for metals from the studies included in this analysis were harmonized. All units were converted to mg/kg for soil and sediment and mg/L for water. This allowed us to easily make comparisons of heavy metal concentrations according to the studies, types of sampling area, countries and continents.

First, the extracted data were analyzed using Microsoft Excel 2013 software. Statistical analyses were done to synthesize the data according to the variables. Second, a narrative synthesis was done on all included studies, and key points on each of the items studied were reported.

## 3. Results and Discussion

### 3.1. Overview

The initial search of titles/abstracts resulted in 3063 published articles, of which 2856 were excluded, because they did not meet the predefined criteria (see Figure 1 PRISMA flowchart). Of the remaining 187 articles that were entirely read, 59 studies were included in this review, with reasons for excluding articles detailed in Figure 1.

The studies included in this analysis spanned 11 countries and were spread across all continents, except Europe. Of the 59 included studies, 71% were conducted in China, 15.3% in Africa and 13.8% in the rest of the world. All the included studies were cross-sectional. Only 15 out of the 59 studies had a reference site. Generally, the studies analyzed metals at e-waste recycling sites, around e-waste recycling sites, in specific dump sites, in free recycling sites and in residential areas. The metals were analyzed in various subcomponents of soil (top, middle, deep, farmland and forest), water (borehole, tap, well, spring, stream and drinking water) and sediment (dust and river). Of the five target metals, only Pb was common to all studies and measured in each (Table 1).

Regarding the instruments used to detect the heavy metals, about one-third (33.8%) of the studies used inductively coupled plasma mass spectrometry, while the rest used atomic absorption spectroscopy (22.1%), inductively coupled plasma atomic emission spectroscopy (16.2%), inductively coupled plasma optical emission spectrometry (14.7%) and atomic fluorescence spectrometry (8.82%).

### 3.2. Soil Contamination

**Figure 2:**
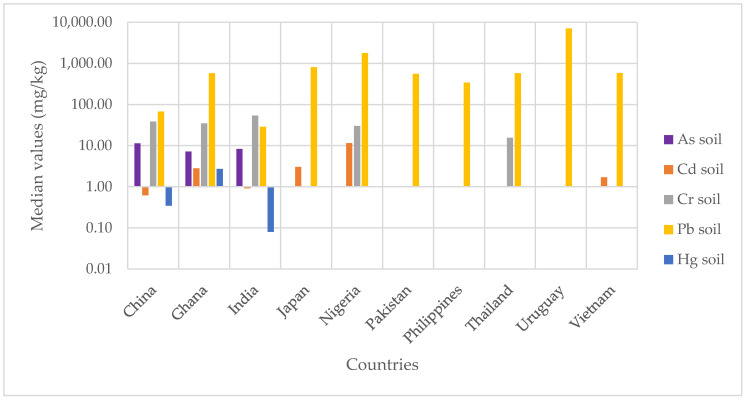
Median concentrations of heavy metals in soil by country.

The median values of the concentrations of heavy metals in the soil by country are summarized in Figure 2. In addition, Table 2 summarizes the middle and upper values of each heavy metal, each sample type and sample size. The Supplemental Materials provide details on sample sizes and middle and upper values of each heavy metal.

#### 3.2.1. Arsenic in Soil

In this group, 14 studies from three countries were identified, in which measurements of As were taken from 595 soil samples. The pooled central median concentration of As in soil was 9.55 mg/kg (see Figure 2) (interquartile range—IQR: 5.45–17.19 mg/kg), with the upper bound median value being 18.95 mg/kg (Table 2 and Figure S1). The highest concentrations were recorded in China, followed by Ghana and India. It should be noted that in the sampling areas, the highest average concentrations of As were found in abandoned recycling sites [34], followed by recycling sites [35,36,37] (see Table 3). However, the highest concentration was identified by the study conducted by Li, Duan and Shi [35]. This may be explained by the fact that there have been many more studies on recycling sites than on abandoned recycling sites, which have only been the subject of one study [34].

Comparing the values of each study with the United States Environmental Protection Agency (USEPA) guidelines [38], it can be seen that only the value recorded in the Wang et al. [39] study, conducted around a recycling site, is below the standard. All other values from other studies are well above the recommendation (see Figure S1). In addition, even studies that have included free recycling sites report values above the standards [36,40], which shows the extent of soil pollution by As from e-waste.

#### 3.2.2. Cadmium in Soil

In this group, 36 studies from six countries were identified. Combined, these studies analyzed 1625 soil samples in total. The pooled central median concentration of Cd in soil was 1.11 mg/kg (Figure 2) (IQR: 0.18–4.81 mg/kg), with the upper bound median value being 2.48 mg/kg (Table 2 and Figure S2). The highest concentrations were recorded in China [41], followed by Ghana [40], India [42], Nigeria [43], Japan [44] and Vietnam [45] (see Figure S2). In fact, almost all studies on Cd and soil have been conducted in China. In the sampling areas, the highest concentrations of Cd in soil were recorded in recycling sites [41,43,46,47,48], followed by abandoned recycling sites [34,46] (see Table 3).

As presented in Figure S2, comparison of the upper values in the literature with the USEPA guidelines shows that, in China, several values are above the recommended limit [34,41,46,47,48,49,50,51]. In addition, several other studies have reported above-standard values in Ghana [40,52,53], India [42] and Nigeria [43].

#### 3.2.3. Chromium in Soil

In this group, 32 studies from five countries were identified. Combined, these studies analyzed 1310 soil samples in total. The pooled central median concentration of Cr in soil was 34.79 mg/kg (see Figure 2) (IQR: 18.97–69.36 mg/kg), with the upper bound median value being 49.3 mg/kg (Table 2 and Figure S3). The highest concentrations were recorded in Ghana [53], followed by China [35], India [42], Nigeria [43] and Thailand [54], in that order. In fact, almost all the studies on Cr and soil have been conducted in China and Ghana (see Figure S3). The high Cr concentrations according to the sampling areas were recorded in abandoned recycling sites [34,46], followed by recycling sites [25,35,48,53,55,56,57] and around recycling sites [22,34,36,55,58,59] (see Table 3).

According to Figure S3, the comparison between Cr values in soil and the USEPA guidelines shows that only the study by Quan et al. [60] reported values below the standard. All other studies reported values well above the recommended limit (Figure S3). It should be noted that even studies that have included non-recycling sites report values above the standard [36,40,58,60,61]. This further shows the extent of soil pollution by Cr from e-waste.

#### 3.2.4. Lead in Soil

In this group, 42 studies from 10 countries were identified. Combined, these studies analyzed a total of 1607 soil samples. The combined central median concentration of Pb in soil was 96.46 mg/kg (see Figure 2) (IQR: 46.88–466.5 mg/kg) with the upper bound median value being 162.5 mg/kg (Table 2 and Figure S4). The highest concentrations were recorded in Uruguay [62], followed by Ghana [52], China [50], Thailand [18], Nigeria [43], India [42], Japan [44], Vietnam [45], the Philippines [23] and Pakistan [63], in decreasing order (see Figure S4). It should be noted that about 2/3 of studies on soil contamination by Pb were conducted in China. Table 3 shows that the high concentrations were recorded in residential areas, even though they were near recycling sites [62], followed by abandoned recycling sites [34,46,64] and recycling sites [10,18,36,40,43,44,65,66].

By comparing the soil Pb upper values reported in the literature and USEPA guidelines, many of them are above the accepted limit. These non-standard values have been found in Uruguay [62], Ghana [10,40,52,53], China [34,36,46,50,64,66], India [42], Japan [44], Nigeria [43], Pakistan [63], the Philippines [23], Thailand [18] and Vietnam [45].

#### 3.2.5. Mercury in Soil

In this group, 42 studies from 10 countries were identified. Combined, these studies analyzed a total of 699 soil samples. The pooled central median concentration of Hg in soil was 0.34 mg/kg (see Figure 2) (IQR: 0.09–2.47 mg/kg), with the upper bound median value being 1.08 mg/kg (Table 2 and Figure S5). It should be noted that the highest concentrations were recorded in China [60], followed by Ghana [52] and India [42]. Almost all studies on soil contamination by Hg have been conducted in China (see Figure S5). As presented in Table 3, the high concentrations of Hg in soil according to the sampling areas were recorded in abandoned recycling sites [34,67], followed by recycling sites [42,53,60,67].

The comparison between the upper values of Hg in soil reported in the literature and the USEPA guidelines shows that several concentrations are above the guidelines (Figure S5). These non-standard concentrations have been recorded in China [34,50,60,67], Ghana [52,53] and India [42]. It should be noted that one study found values above the standard in a free recycling site [67]. This shows the extent of soil pollution by Hg contained in e-waste.

#### 3.2.6. Summary of Studies Concerning Metals in Soil

By synthesizing the information on all heavy metals in soil, we note in this systematic review a predominance of studies conducted in Asia, especially in China, on the contamination of soils by heavy metals from e-waste recycling. The imbalance of the scientific data on the topic does not allow for a global perspective, such that a more or less universal decision may be made or conclusion drawn. It is therefore important to conduct studies in other developing regions so that the analysis is truly global.

In addition, studies on soil contamination by heavy metals are more numerous than studies on other aspects of environmental contamination (water and sediment). This situation could be explained, first, by the position of the soil in relation to recycling activities and the ease with which a scientist can determine the heavy metals in the soil and attribute the concentrations directly to e-waste. Indeed, e-waste is recycled on soil and this direct contact obviously explains the concentrations detected in the soil [35,68]. On the other hand, contamination of water and sediment can take place through infiltration or resuspension of the particles, with many confounding factors to be accounted for by the scientist [69]. In fact, at or near most e-waste recycling sites, there are usually many other human activities (e.g., vehicular traffic, food preparation, biomass burning) that can contaminate ecosystems. Secondly, soil sampling can be done at the surface or at depth with less expensive equipment and simple techniques, while the sampling of water requires specific techniques and equipment, depending on whether it is surface water or groundwater [69].

### 3.3. Water Contamination

**Figure 3:**
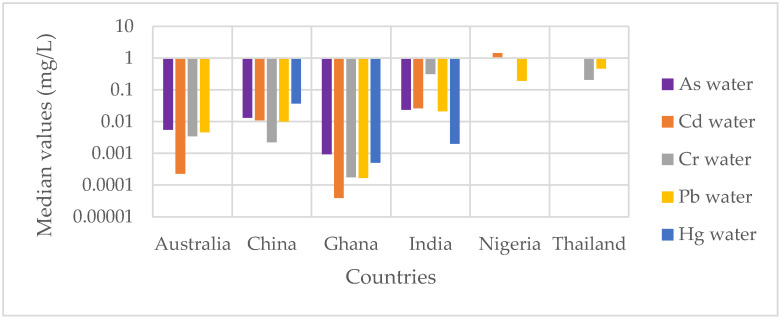
Median concentrations of heavy metals in water by country.

The median values of the concentrations of heavy metals (As, Cd, Cr, Pb and Hg) in water by country are summarized in Figure 3. In addition, Table 2 summarizes the middle and upper values of each heavy metal, each sample type and sample size. The Supplemental Materials provide details on sample sizes and middle and upper values of each heavy metal.

#### 3.3.1. Arsenic in Water

In this group, six studies from five countries were identified. Combined, these studies analyzed 241 water samples in total. The pooled central median concentration of arsenic in water was 0.0064 mg/L (see Figure 3) (IQR: 0.001–0.016 mg/L), with the upper bound median being 0.007 mg/L (Table 2 and Figure S6). The highest concentrations were recorded in China [70], followed by India [56], Australia [71] and Ghana [72] (see Figure S6). Analysis of the values reported in the literature by sampling area shows that the highest concentrations of As in water were recorded in recycling sites [56,70,71] (see Table 3).

Comparing the upper values of As in water with WHO guidelines [73], it can be seen that some concentrations are above the recommended limit (see Figure S6). These limit exceedances were observed in China [70] and India [56].

#### 3.3.2. Cadmium in Water

In this group, 10 studies from five countries were identified. Combined, these studies analyzed 452 water samples. The combined central median concentration of Cd in water was 0.00049 mg/L (see Figure 3) (IQR: 0.00004–0.07 mg/L), with the upper bound median value being 0.00095 mg/L (Table 2 and Figure S7). The highest concentrations were recorded in China [46], followed by Nigeria [74], India [56], Australia [71] and Ghana [72]. As Table 3 presents, analysis of the upper values reported in the literature by sampling area shows that water from around recycling sites [46,74,75] is more contaminated by Cd, followed by recycling sites [11,51,56,70,74]. This paradox can be explained by the fact that a study was carried out around a large recycling site, which was highly contaminated by Cd [46].

Comparing the upper values of Cd in water with the WHO guidelines, it can be seen that some concentrations are above the recommended limit (see Figure S7). These limit exceedances have been observed in China [46,51,70,75], Nigeria [11,74] and India [56].

#### 3.3.3. Chromium in Water

In this group, eight studies were identified in six countries. Combined, these studies analyzed 235 water samples. The pooled central median concentration of Cr in water was 0.0022 mg/L (see Figure 3) (IQR: 0.00023–0.02 mg/L), with the upper bound median value being 0.0028 mg/L (Table 2 and Figure S8). The highest concentrations were recorded in China [46], followed by India [56], Thailand [54], Australia [71] and Ghana [72] (see Figure S8). Analysis of the upper values reported in the literature according to the sampling area shows that water around recycling sites [46,54,72] is more contaminated by Cr, followed by actual recycling sites [54,56,71] (see Table 3). This may be explained by the fact that the study by Luo, Liu, Wang, Liu, Li, Zhang and Li [46] was conducted around a large recycling site heavily contaminated by Cr.

The comparison between the upper values reported in the literature and the WHO guidelines shows that several reported Cr concentrations in water are above the guidelines (see Figure S8). Exceedances have been recorded in China [46], India [56] and Thailand [68].

#### 3.3.4. Lead in Water

In this group, 10 studies from six countries were identified. Combined, these studies analyzed 287 water samples. The combined central median concentration of Pb in water was 0.0063 mg/L (see Figure 3) (IQR: 0.00027–0.227 mg/L). The upper bound median value was 0.01 mg/L (Table 2 and Figure S9). The highest concentrations were recorded in China [46], followed by Nigeria [74], Thailand [54], India [56], Australia [71] and Ghana [68] (see Figure S9). It should be noted that according to the analysis of the upper values reported in the literature, water from around recycling sites [46,54,74,75] is more contaminated with Pb, followed by recycling sites [11,54,56,70,74] (see Table 3). This contrast can be explained by the high concentrations reported in the study by Luo, Liu, Wang, Liu, Li, Zhang and Li [46] which was conducted in a heavily polluted around recycling site.

As shown in Figure S9, the comparison between WHO guidelines and upper Pb values in water has shown concentrations above the standard in several countries. These non-standard concentrations have been recorded in China [46,70,75], Nigeria [11,74], Thailand [54], India [56] and Australia [71].

#### 3.3.5. Mercury in Water

Regarding water contamination by mercury, four studies from three countries were identified. Combined, these studies analyzed 132 water samples. The pooled central median concentration of Hg in water was 0.0005 mg/L (see Figure 3) (IQR: 0.0005–0.00125 mg/L), with the upper bound median value being 0.0005 mg/L (Table 2 and Figure S10). China [67], followed by India [56] and Ghana [72], recorded the highest concentrations (see Figure S10). Analysis of the upper values of Hg according to the sampling areas showed that water from recycling sites [56,70] is the most contaminated with Hg (see Table 3).

Upper values of Hg in water compared with WHO guidelines show that, in China, there are concentrations above the recommended limit [67]. After synthesizing all the information of all heavy metals in water, it is important to point out that the studies on water contamination included in this review were mostly conducted in Asia, especially in China. Research initiatives should therefore be encouraged in other developing regions of the world, such as South America, Southeast Europe and Africa. Indeed, a large and balanced amount of scientific data will undoubtedly provide a more comprehensive view of the adverse effects of e-waste recycling on ecosystems. In addition, the highest concentrations were all recorded in China and, therefore, in Asia. This situation could lead to public health problems. Indeed, heavy metals are bio-accumulative and heat-resistant pollutants [54,76], and as it is known that people continue to use well water in the kitchen and others use it as drinking water [77,78], we wonder about the morbidity associated with e-waste activities. Indeed, several other studies have proven the harmful effects of heavy metals on human health [27,79,80].

### 3.4. Sediment Contamination

**Figure 4:**
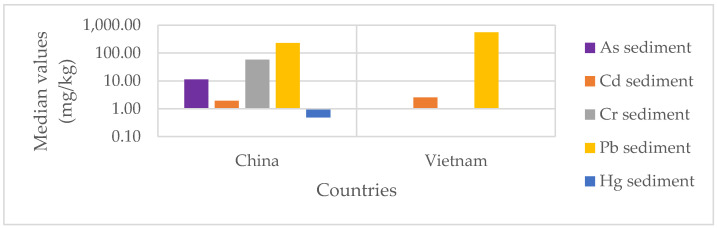
Median concentrations of heavy metals in sediment by country.

The median values of heavy metal concentrations (As, Cd, Cr, Pb and Hg) in sediment by country are summarized in Figure 4. In addition, Table 2 summarizes the middle and upper values of each heavy metal, each sample type and sample size. The Supplemental Materials provide details on sample sizes and middle and upper values of each heavy metal.

#### 3.4.1. Arsenic in Sediment

Only three studies—all conducted in China—were found on the contamination of sediment by As. The studies analyzed a total of 107 sediment samples. The median central pooled concentration of As in sediment was 11.3 mg/kg (see Figure 4) (IQR: 8.02–11.45 mg/kg), with the upper bound median value being 15.55 mg/kg (Table 2 and Figure S11). The highest concentrations were recorded at recycling sites [70]. Thus, recycling sites are the most contaminated, just ahead of free recycling sites [81] (see Table 3). This shows the extent of the effect that contaminated sites can have on the contamination of areas that are not covered by e-waste recycling activities.

It is important to note that all values reported in the literature on As in sediment [39,70,81] are above the limit recommended by the USEPA guidelines (see Figure S11).

#### 3.4.2. Cadmium in Sediment

Twelve studies, conducted in two countries (China and Vietnam), were found on the contamination of sediment by Cd, with 11 of them conducted in China. These studies analyzed 347 sediment samples in all. The combined central median concentration of Cd in sediment was 2.15 mg/kg (see Figure 4) (IQR: 1.01–4.8 mg/kg), with the upper bound median value being 3.52 mg/kg (Table 2 and Figure S12). The highest concentrations were recorded in China [81,82,83], followed by Vietnam [45] (see Figure S12). Analysis of upper Cd values in sediment, by sampling area, showed that recycling sites [45,70,81,82,84,85,86] and areas around recycling sites [39,61,83,87,88] are simultaneously the most contaminated (see Table 3).

By comparing the upper values of Cd in sediment with the USEPA guidelines, all of the studies reported concentrations above the recommended threshold (see Figure S12). It should be noted that even non-recycling sites [61,81,84] have values above the standard, which testifies to the influence of recycling site contamination on areas where e-waste is not recycled.

#### 3.4.3. Chromium in Sediment

Only three studies, all conducted in China, showed Cr contamination in sediment. Combined, these studies analyzed 171 sediment samples. The combined central median concentration of Cr in the sediment was 58 mg/kg (see Figure 4) (IQR: 39.52–67.72 mg/kg), with the upper bound median value being 129.5 mg/kg (Table 2 and Figure S13). Analysis of upper Cr values in sediment, by sampling area, showed that recycling sites [81,82,84,85] are the most polluted, followed by areas around recycling sites [61,83,87] (see Table 3).

As presented in Figure S13, the comparison between the upper values of Cr in sediment and the USEPA guidelines shows that all the studies reported concentrations above the recommended limit. It should be noted that even studies including non-recycling sites [61,81,83,84] have reported Cr concentrations above the limit allowed in sediment, indicating the influence of recycling site contamination on areas where e-waste is not recycled.

#### 3.4.4. Lead in Sediment

Thirteen studies, conducted in two countries (China and Vietnam), were found on the contamination of sediment by Pb, of which 12 were conducted in China. Combined, these studies analyzed 371 sediment samples. The pooled central median concentration of lead in sediment was 233.5 mg/kg (see Figure 4) (IQR: 65.1–528.87 mg/kg), with the upper bound median value being 287 mg/kg (Table 2 and Figure S14). The highest concentrations were recorded in China [89] and then Vietnam [45]. Analysis of upper Pb values in sediment by sampling area showed that recycling sites [45,70,81,82,83,84,85,86,89] are the most polluted, followed by areas around recycling sites [39,61,83,87,89] (see Table 3).

By comparing the upper values of Pb in sediment with the USEPA guidelines, all the studies reported concentrations above the recommended limit (see Figure S14). It should be noted that even non-recycling sites [61,83,84] had values above the standard, which testifies to the influence of recycling site contamination on areas where e-waste is not recycled.

#### 3.4.5. Mercury in Sediment

Only three studies, all conducted in China, reported on Hg contamination in sediments. Combined, these studies analyzed 107 sediment samples. The median central pooled concentration of Hg in the sediment was 0.49 mg/kg (see Figure 4) (IQR: 0.12–0.84 mg/kg), with the upper bound median value being 0.95 mg/kg (Table 2 and Figure S15). The highest concentrations were recorded at recycling sites [70,81]. As presented in Figure S15, the comparison between the upper values of Hg in sediment and the USEPA guidelines shows that the studies by Guo, Huang, Zhang and Dong [70] and Chen, Yu, Shen, Zhang, Liu, Shen, Tang and Chen [81] reported concentrations above the recommended limit. It should be noted that even the study by Chen, Yu, Shen, Zhang, Liu, Shen, Tang and Chen [81], which included free recycling sites, found Hg concentrations above the limit allowed in sediments, indicating the influence of recycling site contamination on areas where e-waste is not recycled.

In synthesizing information on heavy metal pollution in sediments, we noticed that almost all these studies were conducted in China. There is therefore a need for research in other parts of the world for a global analysis of the situation.

## 4. Conclusions

This systematic review analyzed peer-reviewed studies published between the years 2005 and 2017 that reported on As, Cd, Cr, Hg and Pb levels in soil, water and sediment collected from areas that recycle e-waste. In general, there are five broad conclusions of this work. First, across all studies, the concentrations of metals in a given medium are generally higher than international standards. Second, the risks associated with such exposures for both human health and the environment are not well characterized, and, as such, further studies are needed. Third, there are geographical data gaps, as most studies were from Asia (notably China). As e-waste recycling occurs in many countries worldwide, there is a need to increase research in other geographical areas like Africa, Oceania, Europe, America and even in Asian countries other than China. This will allow a clearer comparison between contamination within developing countries (often conducted in the informal sector without regulations) and that of developed countries (with a regulatory framework establishing clear guidelines for recycling, and health and safety rules). Here, we only focused on papers published in English and French, and we acknowledge that studies published in different languages were missing. Fourth, we note that many studies did not include key variables (e.g., exact sampling dates, reference materials, analytical quality control, control sites). A future study of this kind could design a risk of bias score to identify (and better consider) higher quality studies. Fifth, the findings here raise concern about metal contamination at e-waste sites worldwide, and thus it is imperative for actions (e.g., policy changes, engineering solutions, educational campaigns) to be taken at these sites to help reduce exposure (and thus risk).

## Figures and Tables

**Table 1:** Overview of included studies. As detailed in Figure 1, 59 peer-reviewed scientific articles met the criteria for inclusion in this systematic review, and here we provide key metadata for each of these studies. Additional details are in the Supplemental Materials, including a list of all the included articles.

Regions	Countries	Metals Analyzed	Studies	Sample Type and Size	Sample Area
Oceania	Australia	As, Cd, Pb, Cr	1	- Well water (*n* = 372)	- Recycling site - Residential area
Africa	Ghana	As, Cd, Pb, Cr, Hg	5	- Soil (*n* = 716) - water (borehole, stream, spring, tap well) (*n* = 240)	- Recycling site - Around recycling site - Abandoned recycling site - Dumping recycling site - Free recycling site
	Nigeria	As, Cd, Pb, Cr	4	- Soil (top, middle, deep) (*n* = 633) - water (tap, well) (*n* = 132)	
Asia	China	As, Cd, Pb, Cr, Hg, MeHg, T-Hg	42	- Soil (top, middle, deep, farmland, forest) (*n* = 4060) - Water (well, drinking, pond, fresh) (*n* = 505) - Sediment (dust, river) (*n* = 1055)	- Recycling site - Around recycling site - Abandoned recycling site - Dumping recycling site - Free recycling site - Residential area
	India	As, Cd, Pb, Cr, Hg	2	- Soil (*n* = 165) - Well water (*n* = 50)	
	Japan	Cd, Pb	1	- Soil farmland soil (*n* = 40)	
	Pakistan	Pb	1	- Soil (*n* = 3)	
	Philippines	Pb	1	- Soil (*n* = 56)	
	Thailand	Pb, Cr	2	- Soil (*n* = 75) - Water (*n* = 48)	
	Vietnam	Cd, Pb	1	- Sediment (dust) (*n* = 48) - Soil (*n* = 48)	
South America	Uruguay	Pb	1	- Soil (40)	- Residential area
Total	-	-	62 *	-	-

* In Table 1 the total of studies is 62 instead of 59 because 2 studies were both conducted in 2 different countries, thus duplicating a study for 2 countries.

**Table 2:** Summary of measured concentrations (central and upper values) of metals in e-waste sites for soil, water and sediment.

Metals	Soil (mg/kg)			Water (mg/L)			Sediment (mg/kg)		
	*n*	Middle	Upper	N	Middle	Upper	*N*	Middle	Upper
As	595	9.55	18.95	241	0.0064	0.007	107	11.3	15.55
Cd	1625	1.11	2.48	452	0.00049	0.00095	347	2.15	3.52
Cr	1310	34.79	49.3	235	0.0022	0.0028	171	58	129.5
Pb	1607	96.46	162.5	287	0.0063	0.01	371	233.5	287
Hg	699	0.34	1.08	132	0.0005	0.0005	107	0.49	0.95

**Table 3:** Summary of the average central value concentration of certain metals according to specific sample areas in e-waste sites.

Metals	Abandoned Recycling Site	Around Recycling Site	Free Recycling Site	Recycling Site	Residential Area
**Soil** (**mg/kg**)					
As	36.60	7.73	9.89	34.72	0.28
Cd	21.28	0.91	0.27	48.36	0.04
Cr	388.96	34.05	23.34	65.46	16.35
Pb	2221.70	97.86	32.75	729.95	3565.71
Hg	568.03	0.25	50.05	222.71	
**Water** (**mg/L**)					
As	No data	0.01	0.00	0.31	0.01
Cd		0.77	0.00	0.38	0.00
Cr		4.88	0.00	0.30	0.01
Pb		188.19	0.00	0.43	0.01
Hg		0.00	0.00	0.06	
**Sediment** (**mg/kg**)					
As	No data	0.29	6.36	11.39	No data
Cd		3.82	0.98	3.82	
Cr		73.61	43.73	136.19	
Pb		3894.50	82.62	9699.13	
Hg		0.00	0.16	0.81	

## Data Availability

Data from the literature search in this review are available from the corresponding authors (karelhouessionon@yahoo.fr).

## Supplementary Materials

The supplemental data are available at https://www.mdpi.com/1660-4601/18/7/3517/s1. Figure S1: Middle and upper values of soil As concentrations; Figure S2: Middle and upper values of soil Cd concentrations; Figure S3: Middle and upper values of soil Cr concentrations; Figure S4: Middle and upper values of soil Pb concentrations; Figure S5: Middle and upper values of soil Hg concentrations; Figure S6: Middle and upper values of water As concentrations; Figure S7: Middle and upper values of water Cd concentrations; Figure S8: Middle and upper values of water Cr concentrations; Figure S9: Middle and upper values of water Pb concentrations; Figure S10: Middle and upper values of water Hg concentrations; Figure S11: Middle and upper values of sediment As concentrations; Figure S12: Middle and upper values of sediment Cd concentrations; Figure S13: Middle and upper values of sediment Cr concentrations; Figure S14: Middle and upper values of sediments Pb concentrations; Figure S15: Middle and upper values of sediment Hg concentrations.

## Abbreviations

E-waste	Electronic waste
IQR	Interquartile range
U.S. NIEHS	United States National Institute of Environmental Health Sciences
USEPA	United States Environmental Protection Agency
WHO	World Health Organization
